# Prognostic Factors Determining Mortality in Surgical Neonates

**Published:** 2012-01-01

**Authors:** Vivek Manchanda, Yogesh Kumar Sarin, Siddharth Ramji

**Affiliations:** Department of Paediatric Surgery, Maulana Azad Medical College, New Delhi, INDIA; 1 Department of Neonatology, Maulana Azad Medical College, New Delhi, INDIA

**Keywords:** Surgical neonates, Prognosis, Mortality, Scoring systems

## Abstract

Background: To assess the prognosis of surgical neonates at admission and the factors responsible for mortality in neonates.

Material and Methods: A prospective study was conducted in a tertiary level hospital over 15 months and various clinical and biochemical parameters were collected and analyzed using STATA^®^ and SPSS^®^.

Results: On multivariate analysis of 165 neonates, early gestational age, respiratory distress and shock at presentation were the factors of poor prognosis in neonates. The factors could be related to poor antenatal care and sepsis acquired before transfer of the baby to the nursery.

Conclusion: The improvement in antenatal care and asepsis during transfer and handling the babies is of utmost importance to improve the prognosis of surgical neonates.

## INTRODUCTION

Many studies have been conducted in various parts of the world to classify the neonates as to the risk strata soon after delivery or admission especially very low birth weight (VLBW) infants. Among these the most prominent are clinical risk index for babies (CRIB) score, CRIB II, score for neonatal acute physiology (SNAP), score for neonatal acute physiology – perinatal extension (SNAPPE), SNAP II, SNAPPE II, NTISS, National institute of child health and human development (NICHHD) score, the Berlin score and Neonatal mortality prognosis index (NMPI) [1-9]. The above-mentioned scores have been developed and validated on neonates in general.

The only prognostic systems established in surgical neonates are restricted to the Waterston criteria, Montreal classification and Spitz risk grouping in assessing prognosis of child with esophageal atresia with or without tracheo-esophageal fistula, Breaux et al for babies with congenital diaphragmatic hernia and Nixon and Tawes for patients with small bowel atresia [10-14].

The above mentioned scores are disease specific and whether can be generalized to all surgical neonates is debatable. This study was planned to assess the prognostic factors for the surgical neonates and to develop a score for assessing the prognosis of these patients.

## MATERIALS AND METHODS

The study was conducted in Departments of Pediatric Surgery, Neonatology and Pediatrics at our Hospital. Clearance from the ethical committee of the hospital was obtained. The enrollment of patients was started on 1st January 2006 and the last baby was enrolled on 30th March 2007 after taking informed consent. The patients were followed up for minimum of 1 month.

The study was a prospective cohort study. During 15 months of enrollment period, 191 neonates were admitted that required surgical intervention in the institution. Of these 14 babies could not be operated as they succumbed during stabilization period and were excluded from the study. Further, 12 babies could not be included in the study because of non-availability of complete data or absence of parental consent. The standard care of treatment was provided to all neonates. A total of 165 neonates were thus enrolled in the study.

The following clinical and biochemical parameters were recorded and tabulated using Microsoft Excel®: Clinical: Birth and admission weight (Grams), Gestational age (weeks), Heart rate (per minute), Respiratory rate (per minute), Temperature (oF), Blood pressure (mm Hg), APGAR score (at 1, 5 and 10 minute), Seizure (present or absent), Grade of respiratory distress, Urine output (ml/kg/hr) and Associated congenital malformations. Laboratory Parameters: Hematocrit (%), WBC Count (cells/μL), Platelet Count (cells/μL), Blood urea (mg/dl), Serum creatinine (mg/dl), Blood glucose (mg/dl), Serum sodium (mEq/l), Serum Potassium (mEq/l), Serum Calcium (mEq/l), pH, pO2 (mmHg), pCO2 (mm Hg), Base excess (mEq/l), Serum Bicarbonate (mEq/l), Serum Bilirubin – Direct and indirect (mg/dl) and C- Reactive Protein (raised or normal).

All the patients were given treatment as per the protocol of the nursery in which the baby was admitted. The babies were followed up till discharge from the hospital or death. The parents were also instructed for follow up at 1 month after discharge and any adverse events noted. Survival 1 month after surgery was taken as end point for data analysis.

The data was summarized in tabular form and converted to a numbers for statistical analysis. The STATA^®^ and SPSS^®^ were used for the statistical analysis. All the variables were individually tested by parametric and non-parametric tests [either t-test (difference of mean) or chi-square test (difference of proportion)] for calculating statistical association, if any. The factors found significant by difference of means were also dichotomized and the univariate and multivariate analysis was done by logistic regression analysis. The degree of association was calculated by stepwise logistic regression multivariate analysis. However, the new score intended could not be constructed by analyzing the data.

## RESULTS

A total of 107/165 survived for at least one month after surgery and the post-operative mortality was 58/165 (35.15%). A majority of these (27/58) occurred in first 3 days of surgery (46.55%).

The study population was operated for varied indications, majority a congenital malformation as shown in table 1 (Table 1).

**Figure:**
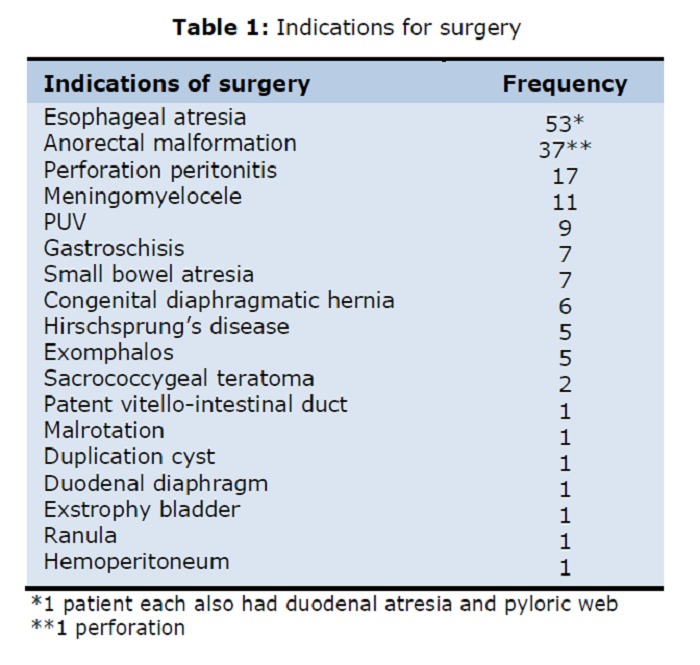
Table 1: Indications for surgery

Demographic Information: The study group was evaluated for difference in age, sex, gestational age and birth weight by difference of means (Table 2). It was found that age and sex (p value greater than 0.05) did not affect the survival although neonates that survived had higher admission weight (p value less than 0.005) and gestational age (p value less than 0.005) than those expired.

**Figure:**
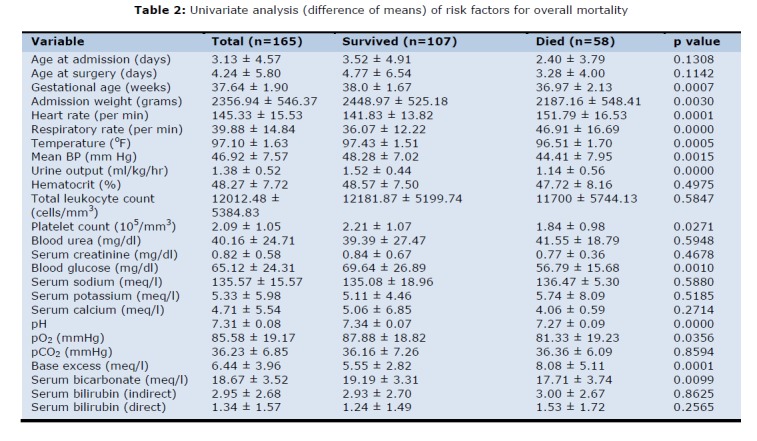
Table 2: Univariate analysis(difference of means) of risk factors for overall mortality

Neonatal Physiologic Alterations: On evaluating clinical parameters in the two groups (survival vs expired) it was found that higher heart rate (p value less than 0.0001), higher respiratory rate (p value less than 0.001), lower temperature (p value less than 0.001), lower blood pressure (p value less than 0.005), presence of respiratory distress (p value less than0.001) and lower urine output (p value less than 0.0001) increased the odds of mortality while occurrence of seizures did not affect survival in neonates (table 2). The APGAR score was available for only 30 patients because the majority of the child births were home deliveries. Thus, we could not assess the role of APGAR score in predicting the mortality in sick neonates. The presence of any congenital malformation other than for the anomaly for which baby was operated upon was recorded. The association could not be quantified, so no statistical association was demonstrated by statistical methods.

Laboratory Parameters: The laboratory values of blood samples drawn at time of admission were evaluated (table 2). Hematocrit (p>0.05), total leucocyte count (p>0.05), deranged kidney function tests (blood urea, serum creatinine, serum sodium and serum potassium) (p>0.05), serum calcium (p>0.05), pO2 (p>0.05) and serum bilirubin (p>0.05) did not predict mortality. On the other hand lower platelet count (p value less than 0.05), lower blood glucose levels (p value less than 0.05), lower pH (p value less than 0.001), higher base deficit (p value less than 0.0001), lower serum bicarbonate (p value less than 0.01) and raised C-reactive protein levels (p value less than 0.001) were found to predict increased mortality in the study cohort.

As the pH, pO2, base deficit and serum bicarbonate values are interdependent, only pH was taken for further analysis. The respiratory distress being dependent upon respiratory rate was also excluded. The cut-off values of normal versus abnormal was taken when the sensitivity approaches the specificity (Table 3). The above mentioned variables were put for further analysis by univariate (difference of proportions) (Table 4) and multivariate analysis using STATA software (Table 5).

**Figure:**
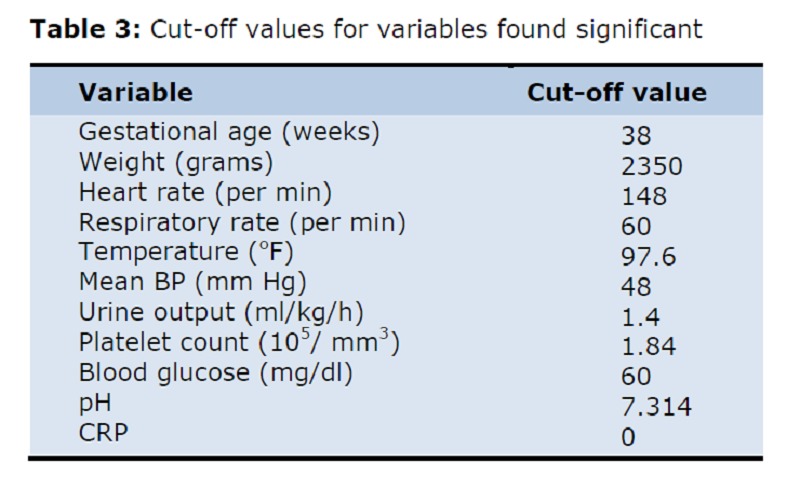
Table 3: Cut-off values for variables found significant

**Figure:**
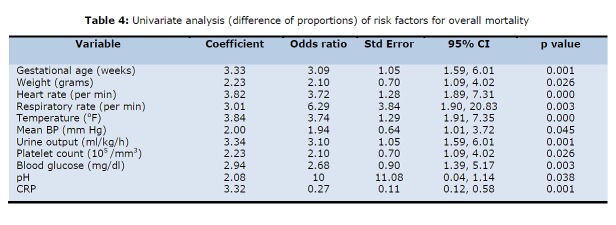
Table 4: Univariate analysis (difference of proportions) of risk factors for overall mortality

**Figure:**
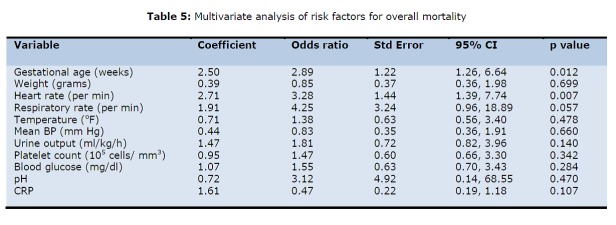
Table 5: Multivariate analysis of risk factors for overall mortality

Thus, after multivariate analysis we could find only gestational age, heart rate and respiratory rate as the independent risk factors that could predict mortality in neonates undergoing surgical intervention. The preterm delivery was associated with increased risk of mortality (3 times increased risk of mortality). Presence of tachycardia at admission (representing stress/ shock) also increased the risk of mortality three-folds. Respiratory distress at presentation was the most important factor in overall mortality increasing the risk of mortality at least 4 times that of population with normal respiratory rate. As only three factors were found predictive, no attempt to construct a score was done.

## DISCUSSION

Illness severity scores are widely used in neonatal care. These scoring systems are useful for Group predictions (Comparing study groups for similarity of risks, Auditing the severity of illness in different units, Comparing performance of different units, Reviewing if infants are treated appropriately for risk, Determining trends in results over time, Comparing rates of complications) and Individual predictions (Giving prognostic information, Stratifying infants in trials, Determining individual treatment) [15].

Usually the scores are created in one of the two ways. “Medical” scores are derived by expert panel using clinical knowledge to select the variables to be included in the score and their relative weights. “Statistical” scores are created when collected data is used in identifying which variables have strong association with the outcome of interest and their relative weights. There is evidence that statistical scores outperform purely medical scores. However, clinical knowledge should contribute to the choice of variables included in the final model [15].

To be practically useful such a score should rely on physiology rather than on diagnosis or therapy. It should be easy to calculate in clinical and research settings, be reliable, have sufficient range to distinguish among the broad spectrum of cases seen in NICUs, be applicable early (at admission), have ability to predict mortality, specific morbidities or cost of various categories of neonates [3].

Various researchers have tried to derive and validate the scores that fulfill the above said criteria to as large an extent as possible. Certain well established of these scores include APACHE and TRISS scores in adults and PRISM and PeRF scores in pediatric intensive care. Neonates are a different set of patients as they have difference in physiology, nature of diseases, associated congenital malformations and different need of care. Thus, these scores were felt insufficient for neonates in general. International Neonatal Network proposed a system of predicting mortality in preterm neonates and small for gestational age babies in 1993. Since then various scoring systems have been proposed for neonates [3,16-19].

Many risk factors have been identified to assess prognosis and to compare the outcome in different nurseries. The traditional risk factors include birth weight, gestational age, respiratory distress at birth, and male sex. That low birth weight is a major determinant of neonatal mortality has been recognized since 1930, when the Finnish pediatrician Yllpo argued that infants born weighing 2500 g or less were at substantially increased risk of death. More recent evidence, however, indicates that birth weight (even in conjunction with other demographic markers such as sex and race) is insufficient to explain large variations in neonatal mortality among neonatal intensive care units [20-22].

The birth weight and gestational age are two markers that have been used widely to classify the neonatal patients in such groups. On univariate analysis in our study, the gestational age and admission weight were significant factors in predicting factors for overall mortality. The relative risk of increase in mortality was three fold for preterm neonates while that was two-fold for low birth weight neonates undergoing surgery. However, on multivariate analysis only the gestational age was significant predictor of overall mortality. This is because of deficient reserves and immunity in preterm neonates. The admission weight could not achieve significance in predicting mortality. This could also point to the fact that the mortality is independent of weight of neonate if the organ systems are mature and proper care is provided to the neonates. The reason could be that the previous studies have been done in VLBW neonates with immature body systems and that has more profound effects on the survival or that in our cohort we included neonates with higher birth weight (only 9 VLBW neonates and 92 low birth weight neonates) with not so much different functional systems, and hence less difference in survival.

The age of the neonate at admission/ surgery have had insignificant effect on mortality as in previous studies. The effect of gender on mortality has been different in studies with most denying any significant association between gender and mortality. Our results also suggested no association between sex of the neonate and mortality.

Among neonatal physiologic parameters, we studied the vitals, respiratory distress and urine output as predictors of mortality. The heart rate signifies hydration status and cardiac function. It may be deranged in any stress, fever, hypothermia, CNS depression secondary to hypoxia or otherwise. Bradycardia was seen in 6 patients at admission of whom 4 (66.7%) died; tachycardia was seen in 18 patients of whom 12 (66.7%) died. The heart rate at presentation was insignificant for early mortality but tachycardia was found to be a significant predictor for overall mortality. It was found that the patients who presented in shock and tachycardia could survive early post-operative by intensive care, ionotropes and in many cases ventilatory support. But the effect of shock persisted and increased overall mortality. The mean blood pressure was found to be significant by univariate analysis but on sequential multivariate analysis could not predict mortality separate from tachycardia. The effect of shock on mortality has been demonstrated by various authors in literature and is also included in the SNAP score.

The respiratory rate (RR) is an indicator of respiratory distress and CNS depression. It may increase in cases of malformed, premature lungs, external compression, increased oxygen demand (like sepsis), pneumonia, etc. It may decrease in cerebral hypoxia or respiratory failure. The respiratory distress was found to be significant in predicting early as well as overall mortality by univariate analysis in accordance with English literature. Two (1.2%) babies were received gasping. Both the babies died. The presence of tachypnea increased the risk of mortality, in the present study, by a factor of 4. This was found to be the most important factor in predicting mortality. The respiratory distress has been included in CRIB scoring system as minimum and maximum FiO2 needed to keep saturation above 85%. Similarly, the SNAP score includes respiratory system immaturity and abnormality as RR, pO2, pO2/FiO2, pCO2 and oxygenation index. Respiratory distress has also found its place in other proposed scoring systems like NICHD score, Berlin score and NMPI score. In 1990, Tarnow Mordi et al found increased sensitivity with inclusion of FiO2 and pH in assessing prognosis of VLBW neonate. The importance of respiratory decompensation as strong predictor of neonatal mortality was identified as early as 1962 by Waterston et al in neonates with esophageal atresia [10,23].

The documentation of hypothermia was also found to be statistically significant in predicting overall mortality by univariate analysis. But hypothermia at presentation lost its statistical significance for overall mortality by multivariate analysis. This suggests that the effect of hypothermia wanes in long term and good neonatal care to keep baby warm can prevent late mortality.

Most of the patients included in the study were out-born, among which most were home deliveries. Thus accurate documentation of APGAR score was not available. Although the previous studies have shown APGAR score to be significant factor in neonatal mortality, we could not evaluate the factor for the same.

The decreased urine output in immediate post-operative period is suggestive of inadequate hydration status, presence of shock, poor renal perfusion and multi-organ dysfunction syndrome in sepsis. The urine output was shown to be a significant factor in predicting overall mortality by univariate analysis. However, the urine output could not achieve significance by multivariate analysis. This can be because oliguria seen in cases with shock/ multi-organ dysfunction syndrome affects mortality while that due to hypovolemic shock can be improved with adequate resuscitation.

The presence of associated life threatening anomalies is significant predictor as these anomalies (mainly cardiac anomalies) can cause death unrelated to the anomaly for which the neonate has been operated. Such decompensation is precipitated by anesthesia and surgical stress. The exact role cannot be defined as the presence could not be exactly quantified.

The hematocrit and total leukocyte count at presentation could not achieve statistical significance. This is expected as the neonate has normal hematocrit even in presence of severe anomalies and maternal anemia. Hematocrit has been included only in SNAP scoring and is removed from SNAP II derived for purpose of simplifying the SNAP score. Lack of statistical significance for TLC may be explained by the delay in the host immune response to antigen thus no significant difference in TLC at presentation. TLC is not included in any score other than SNAP. The platelet count achieved significance by univariate analysis in predicting overall mortality, but the significance was not demonstrated by multivariate analysis.

The renal function at presentation in terms of serum values of blood urea, serum creatinine and serum electrolyte values did not demonstrated any significant difference in cohorts that died and survived. This may be explained by the fact that neonatal blood levels may be within normal limits till 48 hours of age due to feto-placental exchange of factors till birth and slow build up of these in blood over time. They thus are not good predictors of mortality in first 48 hours of life.

Hypoglycemia is considered a very important factor in neonatal care. Hypoglycemia is one of the prognostic markers included in SNAP and is also recognized as an important cause of neonatal mortality in nurseries. We documented significance in predicting mortality by univariate analysis but the association was not found by multivariate logistic regression analysis.

This is understandable as all the neonates undergoing surgical intervention were given continuous infusion of intravenous fluids containing glucose for a considerable period and thus preventing chances of hypoglycemia while in hospital stay. Also, the preterm and VLBW neonates, who are prone to repeated hypoglycemia, were very few in numbers.

Metabolic acidosis as documented by arterial blood gas analysis (pH, base excess, and serum bicarbonate levels) were found to be significant factors in predicting early and overall mortality. The metabolic acidosis occurs in neonates by tissue hypo-perfusion, ischemia resulting in anaerobic metabolism with lacto-acidosis.

The liver function tests as documented by serum bilirubin levels did not statistically differentiate between the neonates who died from those that survived. This is expected as the liver is immature in almost all neonates and the serum bilirubin levels were increased in both the cohorts.

C-reactive protein is a marker for neonatal sepsis. The exact levels could not be documented due to unavailability of standard laboratory. The raised levels were shown to be significant in predicting overall mortality by univariate analysis that lost significance in multivariate analysis. This is against expected as neonatal sepsis is the major cause of mortality in our study population and also shown by other studies previously. This can be explained by the fact that levels of CRP may not have risen at the admission and sepsis is acquired later. The finding may also be biased due to unavailability of standard laboratory in our hospital.

The overall mortality is predicted by shock, respiratory distress and gestational age. The effect of premature respiratory and renal systems was not seen to influence the survival, because the majorities were either full term or borderline preterm.

The improvement requires good antenatal care so as to reduce the chances of pre-term deliveries and better supportive management of these babies. The shock and respiratory distress point directly or indirectly to neonatal sepsis as the most important cause for mortality. The effect of sepsis on respiratory, cardiac and renal function was evident and related directly to the pre-mortality incidents. The importance of hand hygiene and maintenance of strict asepsis in nursery cannot be overemphasized. It was observed, if proper antibiotic if started in proper dosage and at proper time (before onset of irreversible damage), the neonate could be salvaged in most circumstances. Impact of proper supportive care could also be identified in the study group.

We, in the present study, could not derive enough statistical relation to derive any scoring system universally applicable to all surgical neonates. We believe that the variation in derangement of physiology and predisposition to sepsis depends on surgical condition and hence deriving at any reliable scoring system for all surgical neonates may not be possible. Although a larger study may disprove the belief and further studies are needed.

## CONCLUSION

The mortality is high in nurseries especially those catering to surgical patients. The increased mortality can be predicted in preterm neonates. A factor that can improve the outcome in the nurseries in general and specially so in surgical neonates is strict asepsis while handling babies and early diagnosis and treatment of localized and systemic infections. The importance of adequate hand hygiene and its cost-effectiveness in management of neonates and improved outcome cannot be overemphasized.

## Footnotes

**Source of Support:** Nil

**Conflict of Interest:** None declared

